# Identification of hub genes and pathways in lung metastatic colorectal cancer

**DOI:** 10.1186/s12885-023-10792-8

**Published:** 2023-04-06

**Authors:** Wei Dai, Caiyao Guo, Yu Wang, Yumei Li, Renjian Xie, Junhong Wu, Baole Yao, Dong Xie, Ling He, Yingying Li, Hao Huang, Yun Wang, Shenglan Liu

**Affiliations:** 1 School of Pharmacy, Gannan Medical University, Ganzhou, 341000 China; 2 School of Rehabilitation Medicine, Gannan Medical University, Ganzhou, 341000 China; 3 Key Laboratory of Biomaterials and Biofabrication in Tissue Engineering of Jiangxi Province, Gannan Medical University, Ganzhou, 341000 China; 4 Scientific Research Center, The Seventh Affiliated Hospital, Sun Yat-Sen University, Shenzhen, 518107 China

**Keywords:** Colorectal cancer, Differentially expressed genes, Bioinformatics analysis, Hub gene, SFTPD

## Abstract

**Background:**

Colorectal cancer (CRC) is one of the most prevalent types of malignant tumours. Metastasis is the leading cause of cancer-related mortality, with lung metastases accounting for 32.9% of all metastatic CRCs. However, since the biological mechanism of lung metastatic CRC is poorly understood, limited therapeutic targets are available. In the present study, we aimed to identify the key genes and molecular processes involved in CRC lung metastasis.

**Methods:**

The differentially expressed genes (DEGs) between primary and lung metastatic CRC patients were obtained from the Gene Expression Omnibus (GEO) database via the GEO2R tool. The enriched biological processes and pathways modulated by the DEGs were determined with Gene Ontology (GO), Kyoto Encyclopedia of Genes and Genomes (KEGG), and Reactome Gene Sets analyses. The search tool Retrieval of Interacting Genes (STRING) and Cytoscape were used to construct a protein–protein interaction (PPI) network among DEGs.

**Results:**

The DEGs were enriched in surfactant metabolism, cell–cell communication and chemokine signaling pathways. The defined hub genes were included *CLU*, *SFTPD*, *CCL18, SPP1*, *APOE, BGN* and *MMP3.* Among them, *CLU*, *SFTPD* and *CCL18* might be associated with the specific lung tropism metastasis in CRC. In addition, the expression and prognostic values of the hub genes in CRC patients were verified in database of The Cancer Genome Atlas (TCGA) and GEO. Moreover, the protein levels of the hub genes were detected in primary and lung metastatic CRC cells, serum or tissues. Furthermore, SFTPD was confirmed to facilitate cellular proliferation and lung metastasis in CRC.

**Conclusion:**

This bioinformatics study may provide a better understanding of the candidate therapeutic targets and molecular mechanisms for CRC lung metastasis.

**Supplementary Information:**

The online version contains supplementary material available at 10.1186/s12885-023-10792-8.

## Introduction

Colorectal cancer (CRC) is one of the most common and prevalent malignant cancers with the third highest incidence frequency and the second highest mortality rate among all cancers worldwide [1, 2]. In 2022, 151,030 new CRC cases and 52,580 CRC-related deaths were estimated to have occurred in the United States [3]. Approximately 90% of patients with primary CRC cases at early stage can be cured by surgical resection. However, most patients with CRC are diagnosed at advanced stages with recurrence in distant organs, and thus do not have the opportunity to undergo radical surgery [4].

Metastasis is the predominant cause of CRC patient death. According to a recent study, 20% of CRC patients who are newly diagnosed have metastatic disease, and 25% of people with localized CRC will eventually develop metastases. Fewer than 20% of metastatic CRC patients survive for five years [5]. In fact, the lungs are the second most prevalent location of CRC metastasis, accounting for approximately 20–30% of cases [6]. However, limited therapeutic methods are available due to the lack of understanding in the biology of colorectal lung metastases. Therefore, a better understanding of the molecular mechanism of lung metastatic CRC is urgently needed to improve existing treatments and reduce CRC patients’ mortality.

Previous studies have demonstrated that a number of different molecules participate in the development of CRC metastases. For instance, CXCL12/CXCR4, the chemokine receptor pairs, are thought to be associated with liver metastasis and tumour recurrence in CRC [7]. CXCR7 activation is thought to promote the spread of CRC cells to the lung instead of the liver [8]. In addition, some genetic changes, such as WNT pathway activation and RAS mutation, may be linked to an increased proportion of lung metastases [9, 10]. However, these results are scarcely sufficient to provide a comprehensive picture of CRC lung metastases.

Recently, bioinformatics analyses emerged as an efficient and promising tool to screen significantly aberrantly expressed genes and genetic pathways involved in carcinogenesis, which could provide a rationale to identify potential therapeutic targets cancer and understand a cancer prognosis [11–13]. In particular, many studies utilized integrated microarrays analysis and reported that certain vital genes or pathways potentially are involved in CRC liver metastasis or lymph node metastasis [12, 13]. However, studies were quite limited in CRC lung metastases. In this study, the GEO2R tool was utilized to identify differentially expressed genes (DEGs) between primary CRC and lung metastatic CRC tissues based on the GSE41258 and GSE68468 profiles. Subsequently, Gene Ontology (GO), Kyoto Encyclopedia of Genes and Genomes (KEGG) and Reactome Gene Sets analyses were conducted to uncover enriched top biological processes and pathways regulated by the DEGs. The top 10 hub genes related to lung metastasis in CRC and the protein–protein interaction (PPI) network were identified using the search tool Retrieval of Interacting Genes (STRING) and Cytoscape. In addition, the expression and prognostic values of these the hub genes in CRC patients were validated by analyzing the database of The Cancer Genome Atlas (TCGA) and Gene Expression Omnibus (GEO). Furthermore, *SFTPD*, one of the hub genes specifically upregulated in lung metastatic CRC, was validated to promote cellular proliferation and lung metastasis in CRC in vitro and in vivo. In conclusion, the present study may contribute to identifying key genes and pathways for the diagnosis and prognosis of CRC patients with lung metastases, as well as yield novel and viable therapeutic targets.

## Material and methods

### Microarray data

The expression datasets GSE41258, GSE68468, GSE35144, GSE12945, GSE17537, GSE29621, GSE17536 and GSE38832 were obtained from the GEO database (https://www.ncbi.nlm.nih.gov/geo/). GSE41258 dataset includes 378 clinical CRC samples, containing 186 primary CRC and 20 lung metastases. GSE68468 dataset includes 386 clinical CRC samples, containing primary 189 colon tumors and 20 lung metastatic samples.

### Identification of DEGs

The GEO2R online tool (https://www.ncbi.nlm.nih.gov/geo/geo2r/), an interactive web tool, was used to identify DEGs between primary CRC and lung metastatic CRC tissues as previously described [14]. DEGs were designated based on an adjusted *P* value < 0.05 and |log_2_ (fold change)|(log_2_FC) > 1. Heatmaps of the expression of DEGs were acquired using TBtools. The volcano plot of gene expression was established with Graphpad Prism 8. The Venn diagram was analyzed with a web tool (bioinformatics.psb.ugent.be/webtools/Venn/).

### GO/KEGG/ Reactome Gene Sets enrichment analysis

For Gene Ontology (GO) enrichment analysis, DAVID, an online functional annotation tool (https://david.ncifcrf.gov), was applied. For KEGG, which was developed by Kanehisa Laboratories [15], and Reactome Gene Sets were analyzed with Metascape (https://metascape.org/gp/index.html#/main/). A *P* value < 0.05 was the cut-off criterion.

### Protein–protein interaction analysis

The STRING web tool (https://cn.string-db.org) with the default parameters (medium confidence of interaction score) was used to evaluate the potential protein–protein interaction (PPI) relationships among the DEGs. The PPI network was constructed using Cytoscape software (http://www.cytoscape.org/) and visualized by STRING. The molecular complex detection (MCODE) plug-in in Cytoscape was used to extract the modules of the PPI network with the default settings (the degree cut-off = 2, node score cut-off = 0.2, K-core = 2, and max depth = 100).

### Definitions of hub genes

Based on the information from the STRING protein query and degree analysis of the PPI with the cytoHubba plug-in in Cytoscope, we selected the top 10 most dysregulated genes as the hub genes.

### Association between expression levels of hub genes and tumour stage in CRC patients

Based on data from The Cancer Genome Atlas (TCGA) database, the UALCAN web tool (http://ualcan.path.uab.edu/index.html) was used to analyze the correlation between the expression levels of hub genes and the tumour stage of patients with CRC.

### Survival analysis in CRC patients

Based on the information from the GEO database*,* Kaplan–Meier survival analyses for overall survival in CRC patients were performed utilizing Graphpad Prism 8.0. The patients with CRC were divided into two subgroups on the basis of the median expression level of the hub genes.

### Human CRC tissue samples

Informed consent was obtained from individuals or individuals’ guardians following to institutional policies and the Declaration of Helsinki principles. And, pairs of primary and lung metastatic CRC tissues or serum were collected from patients at Gannan Medical University's First Affiliated Hospital and subjected to Western blotting or ELISA assay.

### Cell culture

MC38 cells were obtained from the American Type Culture Collection (ATCC, Manassas, VA) and maintained in RPMI 1640 containing 10% FBS with 1% penicillin–streptomycin (Solarbio, Beijing, China).

### Generating highly lung-metastatic CRC Sublines

MC38 cells (5 × 10^5^) stably expressing luciferase (MC38-Luc) were injected into the tail vein of C57BL/6 mice. Two weeks later, a single nodule on the lung surface was purified and cultured, which was termed as lung metastatic derivatives (MC38-Luc-LM).

### Enzyme-linked immunosorbent assay (ELISA)

As previously described [16], the protein levels of CCL18 were examined in the cell culture medium of MC38-Luc or serum of patients with CRC using ELISA Kit for mice (Cloud-Clone Corp, MEB522Mu) or humans (BOSTER, EK0686). Each sample was measured in duplicate. The median values were employed for the final statistical analysis.

### Western blotting

Cell or tissue lysates were prepared in RIPA buffer containing a protease inhibitor cocktail (Roche, Indianapolis, IN) and separated by SDS-PAGE. The blots were partially cut prior to incubation with antibodies. The following antibodies from Proteintech were used for Western blotting: Clusterin (12,289–1-AP), SFTPD (11,839–1-AP), Osteopontin (22,952–1-AP), MMP3 (17,873–1-AP), APOE (18,254–1-AP), Biglycan (16,409-AP-1) and β-actin (66,009–1-Ig).

### Generation of stable cell lines

The construct encoding mouse SFTPD was cloned into the pTSB-Flag-puro lentiviral vector. Viral supernatants were harvested at 48 and 72 h after transfection with 293 T cells utilizing pCMV-dR8.2 and pCMV-VSVG. MC38-Luc cells were infected with lentiviral supernatants and selected with 1.0 µg/mL puromycin for 5 days to generate stable cell lines.

### Cell Proliferation Assay

For the cell proliferation assay, stable SFTPD-overexpressing cells were seeded in 24-well plates (1 × 10^4^ cells per well). Cell numbers in triple wells were counted with trypan blue staining daily for 6 days.

### Anchorage-independent growth assay

A two-layer soft agar system was used to evaluate the colony formation ability of SFTPD-overexpressing CRC cells according to a previous study [17]. In brief, RPMI 1640 growth medium supplemented with 1% agar and 10% FBS were employed for the first layer, and 10, 000 cells contained in RPMI 1640 medium with 0.5% agar and 10% FBS were used for the second layer. After incubation for ten to fourteen days at 37 °C in a humidified incubator, the colonies (containing more than 50 cells) were counted using an inverted phase-contrast microscope.

### Wound-healing scratch assay

Stable SFTPD-overexpressing MC38-Luc cells (8 × 10^5^ cells/well) were plated into 6-well plates. After the cells reached 100% confluence, a straight wound was created using a 200 μL pipette tip. Then PBS was used to remove the debris and replaced with 1640 medium containing 1% FBS. Images at the indicated times were photographed at 0, 12, 24 and 48 h with a phase contrast microscope.

### Migration and invasion assays

For the migration assay, stable SFTPD-overexpressing MC38-Luc cells were resuspended in FBS-free 1640 medium and seeded into the Transwell inserts (Corning, NY, USA) without Matrigel (Corning, NY, USA). For the invasion assay, cells were resuspended in FBS-free 1640 medium and seeded into the Transwell inserts precoated with 10% Matrigel. Migrated or invaded cells were fixed with 4% paraformaldehyde and stained in 0.1% crystal violet for 10 min after incubation for 24 or 48 h. Three random fields of cells were photographed and counted.

### Lung metastatic mouse model

MC38-Luc cells (5 × 10^5^) were injected into the tail vein of male C56 BL/6 mice aged four-six weeks (GemPharmatech, Jiangsu, China), five mice each group. The in vivo bioluminescence imaging (BLI) was used to examine photon flux in the lung zone of mice. At the end of the experiments, mice were scarified and lungs were resected for BLI, followed by Bouin’s solution fixation for 7 days. H&E staining was conducted as previously reported [18].

### Quantitative real-time PCR (qRT-PCR)

According to the manufacturer’s instructions, Trizol (TransGen Biotch, Beijing, China) was used to extract the total RNA from CRC cells or lung tissues. Subsequently, one-step RT Kit (Thermo Fisher, Shanghai) was used for RNA reverse-transcribed into cDNA. The qRT-PCR reaction was conducted using a BioRAD Real-Time PCR System (Hercules, CA, USA). The qRT-PCR primers are listed in Table S1.

### Statistical analysis

The data analyses were managed using GraphPad Prism software and presented as the means ± SD. Before comparison for significant differences, the normality test was conducted. For normally distributed data, two-tailed Student’s* t* test was used for two-group comparisons and one-way ANOVA, post hoc intergroup comparison was used for comparisons of multiple groups. For non-normally distributed data, Wilcoxon signed-rank test was used for two-group comparisons and the Friedman test was used for comparisons of multiple groups. The log-rank test was used for Kaplan–Meier survival analysis. A *P* value < 0.05 was considered statistically significant.

## Results

### Identification of differentially expressed genes between primary and lung metastatic CRC tissues

**Fig. 1:**
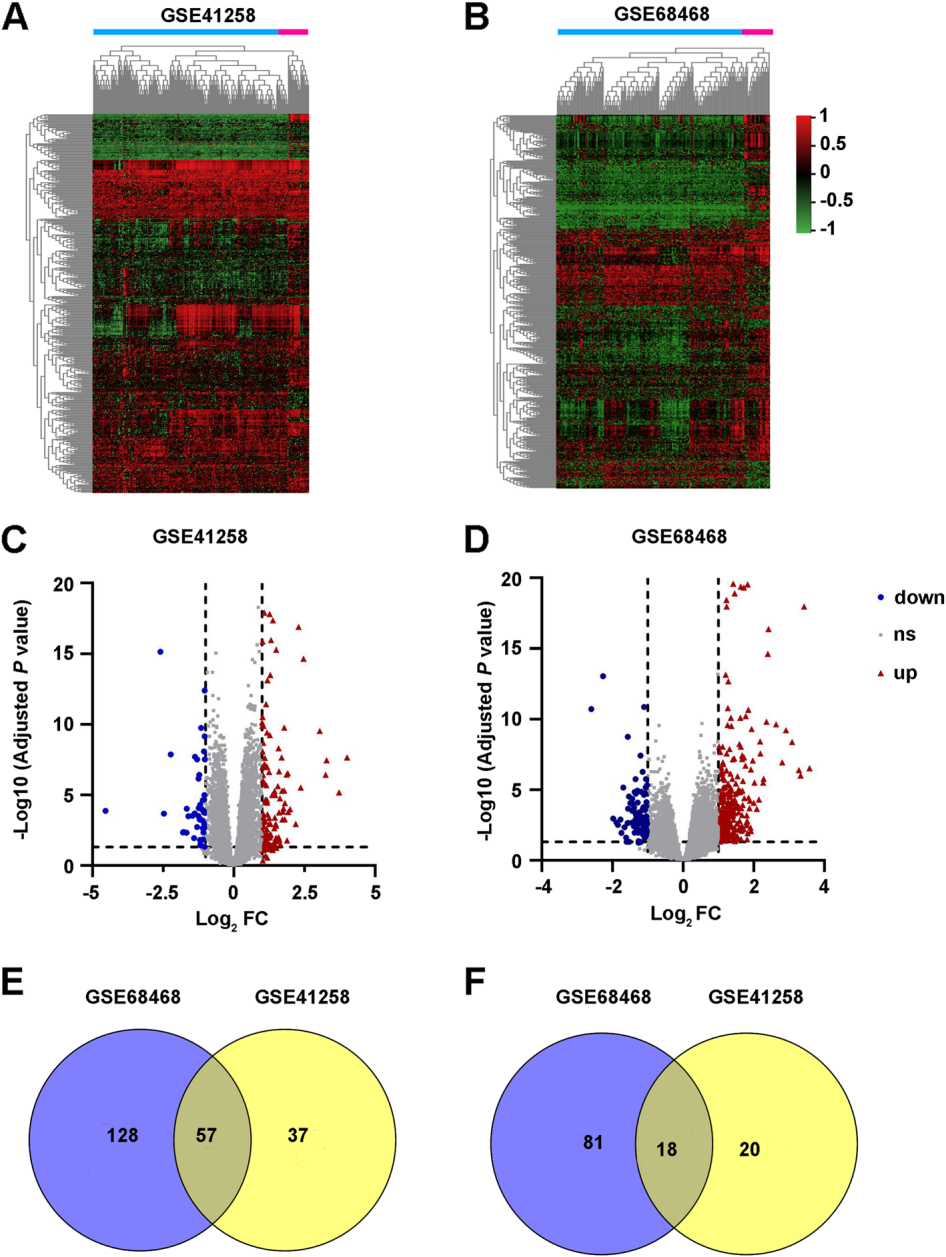
Differentially expressed genes (DEGs) between the lung metastatic and primary colorectal cancer (CRC) samples in the GEO datasets. **A**, **B** Heatmap analysis of DEGs in GSE41258 (**A**) and GSE68468 (**B**). **C**, **D** Volcano plots show the DEGs in GSE41258 (**C**) and GSE68468 (**D**). **E**, **F** Venn diagrams show the intersecting upregulated (**E**) or downregulated (**F**) DEGs in the two datasets

The profiles of GSE68468 and GSE41258 were separately analyzed by the online software GEO2R to screen differentially expressed genes (DEGs) between primary CRC samples and lung metastatic CRC samples. Using |log_2_ (Fold change)|> 1 and the adjusted *P* value < 0.05 as the cutoff criteria, the DEGs in GSE68468 and GSE41258 are screened and shown in Fig. 1A-D. In addition, 57 overlapping upregulated DEGs (185 in GSE68468 and 94 in GSE41258) and 18 overlapping downregulated genes (99 in GSE68468 and 38 in GSE41258) were identified (Fig. 1E, F and Tables S2 and 3). The top 15 significantly overlapping upregulated and downregulated genes were listed in Tables 1 and 2.

**Table 1:** The top 15 upregulated DEGs in GSE41258 and GSE68468 dataset

Gene Symbol	Gene ID	Log_2_ FC in GSE41258	Log_2_ FC in GSE68468	Adj *P* Value in GSE41258	Adj *P* Value in GSE68468
SFTPC	6440	8.332282	5.931846	1.36E-136	3.59E-71
SFTPB	6439	6.479101	6.124719	1.37E-84	1.54E-66
SFTPD	6441	6.04608	4.839111	3.35E-65	1.77E-58
SFTPA2	729,238	5.791667	6.19483	1.57E-64	3.69E-68
SCGB1A1	7356	2.472569	3.36777	2.23E-15	1.10E-24
LTF	4057	2.291725	4.128607	1.29E-17	4.94E-27
CCL18	6362	1.99271	2.289755	0.000103	2.70E-04
IGHM	3507	1.929456	2.133997	0.000159	7.28E-05
CYP1B1	1545	1.908802	2.235139	2.86E-07	3.94E-06
IGLC1	3537	1.887292	1.940792	0.0165	2.18E-02
C7	730	1.870994	2.356968	3.66E-07	8.48E-07
OR7A10	390,892	1.812084	1.372338	3.78E-05	1.05E-05
IGLL1	3543	1.802049	1.054633	0.000541	2.68E-04
ABCA3	21	1.785952	1.532182	1.71E-10	1.03E-06
SLC34A2	10,568	1.774729	2.823726	2.42E-33	2.52E-35

**Table 2:** The top 15 downregulated DEGs in GSE41258 and GSE68468 dataset

Gene Symbol	Gene ID	Log2 FC in GSE41258	Log2 FC in GSE68468	Adj P Value inGSE41258	Adj P Value in GSE68468
MAB21L2	10,586	-2.5946	-2.17959	7.00E-16	1.34E-11
SPINK4	27,290	-2.47337	-1.77526	0.000208	4.38E-03
MMP3	4314	-2.23088	-2.44694	1.34E-08	3.36E-08
MUC2	4583	-1.79232	-1.61319	0.00453	2.23E-02
PLA2G2A	5320	-1.77058	-1.55786	0.00415	8.60E-03
ZG16	653,808	-1.66785	-1.52806	0.00478	3.30E-02
CXCL14	9547	-1.60283	-1.28666	0.000317	4.96E-02
NMU	10,874	-1.45997	-2.03069	0.000296	1.06E-03
PCK1	5105	-1.40534	-1.17076	0.0113	5.76E-02
FUT6	2528	-1.37873	-1.42724	0.000207	1.02E-02
ACTG2	72	-1.29196	-1.23175	0.00191	6.60E-03
ADAMDEC1	27,299	-1.25617	-1.0073	0.000316	1.99E-02
BAX	581	-1.24235	-1.58579	6.89E-07	6.11E-10
GREM1	26,585	-1.20482	-1.18975	0.000545	0.00259
CXCL5	6374	-1.17421	-1.4844	0.0379	5.76E-02

### Functional enrichment analyses

**Fig. 2:**
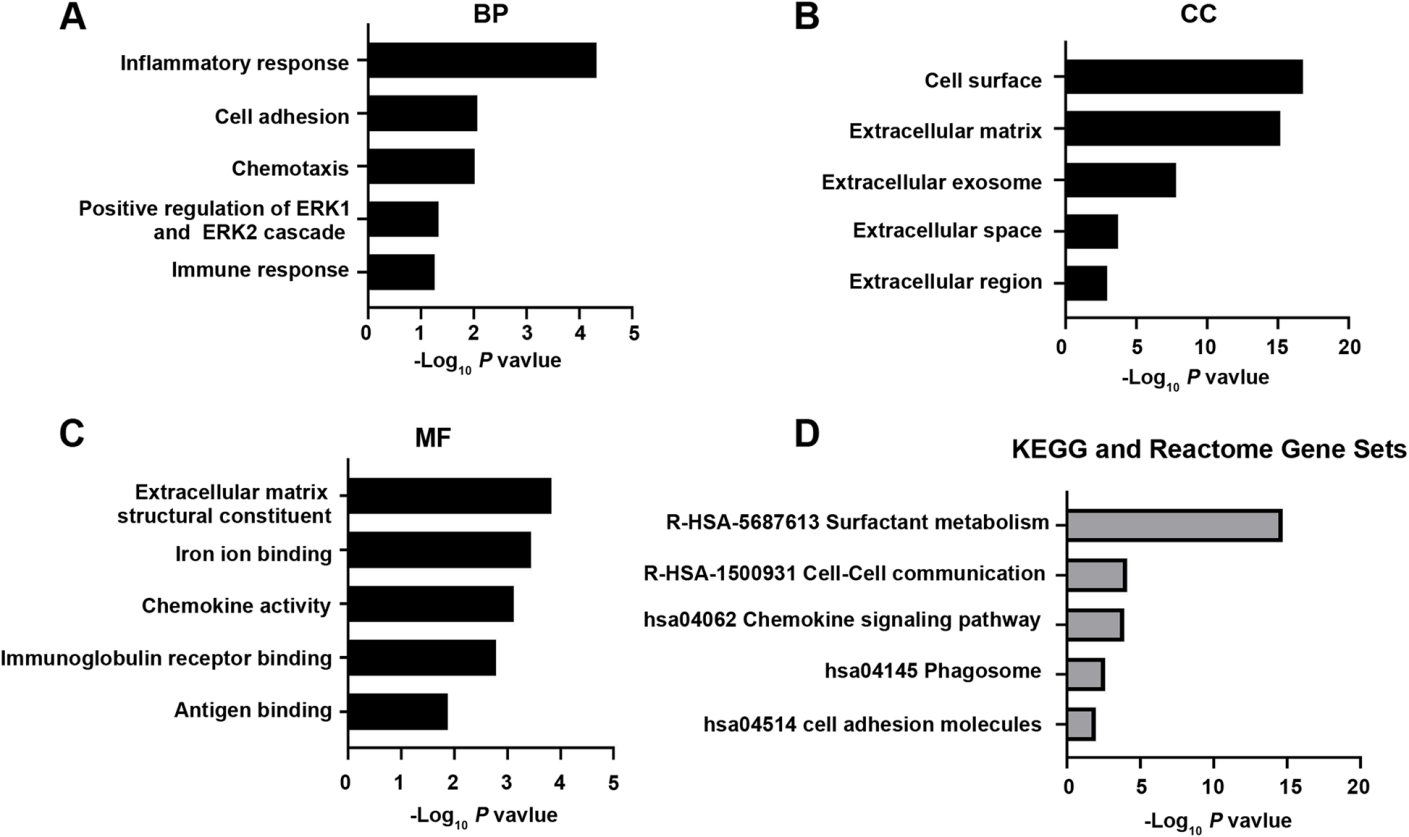
GO, KEGG pathways and Reactome Gene Sets enrichment analyses of differentially expressed genes (DEGs) between primary and lung metastatic colorectal cancer (CRC). **A**-**C** The top 5 biological process (BP), cellular component (CC), and molecular function (MF) categories of the DEGs between primary CRC and lung metastatic CRC used with GO enrichment analysis. **D** KEGG pathways and Reactome Gene Sets enrichment analyses of the DEGs between primary CRC and lung metastatic CRC

GO category enrichment were analyzed by using DAVID soft and the top five significant terms are shown in Fig. 2A-C. DEGs between primary and lung metastatic CRC patients were mainly involved in biological processes (BP) of inflammatory response, cell adhesion, chemotaxis, positive regulation of ERK1 and ERK2 cascade and immune response. For cell component (CC), DEGs were found to be enriched in the formation of cell surface, extracellular matrix (ECM), extracellular exosome, extracellular space and extracellular region. In addition, molecular function (MF) included ECM structural constituent, iron ion binding, chemokine activity, immunoglobulin receptor binding and antigen binding.

KEGG and Reactome Gene Sets analyses were next performed and these DEGs were enriched in cell adhesion molecules, phagosome, chemokine signaling pathway, cell–cell communication and surfactant metabolism (Fig. 2D).

### Hub gene identification, protein–protein interaction (PPI) network construction, and module analysis

**Fig. 3:**
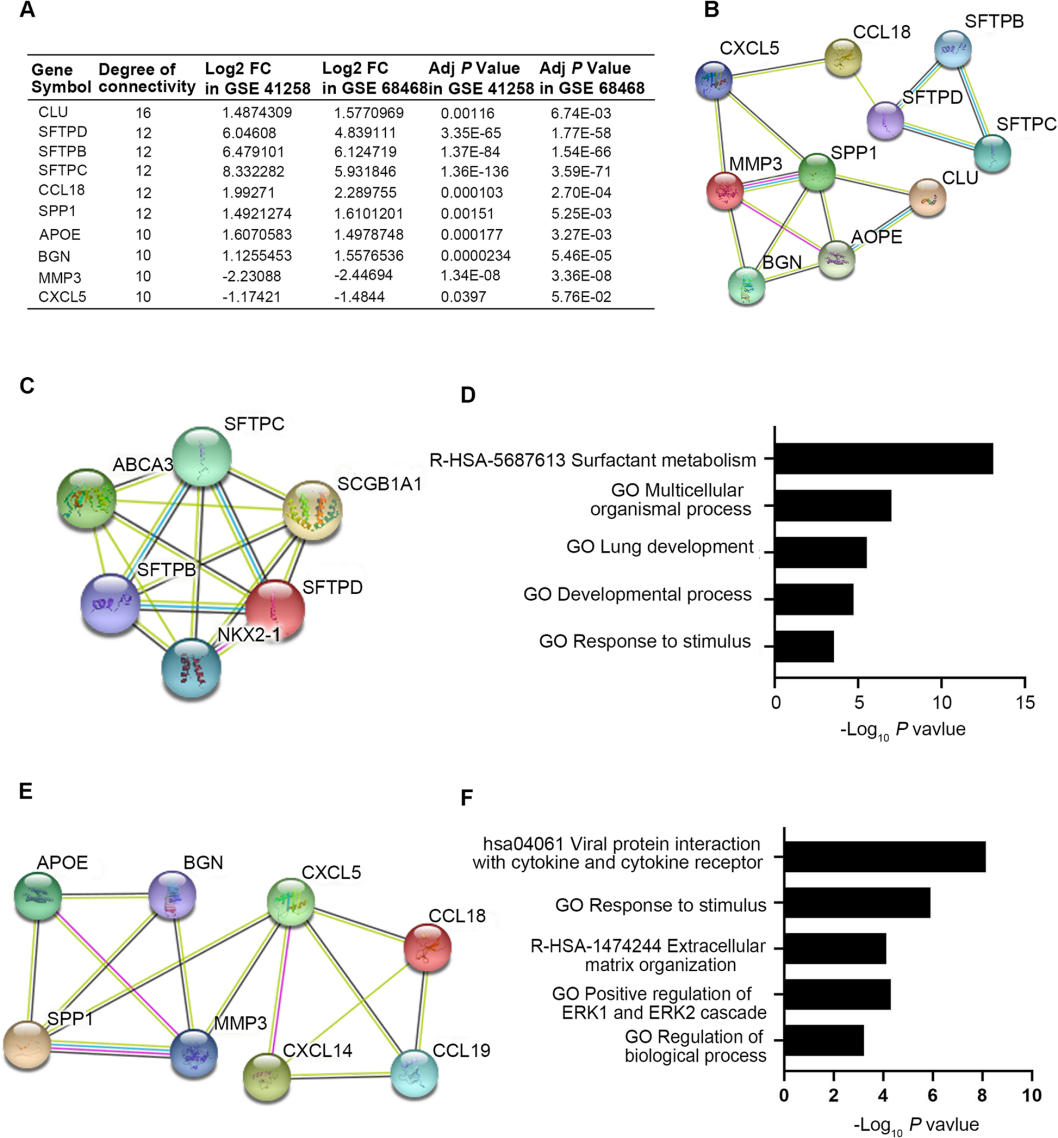
The top 10 hub differentially expressed genes (DEGs) and the protein–protein interaction (PPI) network of the top two modules between primary and lung metastatic colorectal cancer (CRC). **A** Ten hub DEGs with the highest degrees of connectivity between primary and lung metastatic CRC were obtained by STRING and analyzed in Cytoscape. **B** The PPI network of the top 10 hub genes was visualized by STRING database. **C**, **D** Module 1 **C** and the correlated enriched GO, KEGG pathways and Reactome Gene Sets **D** were analyzed with Cytoscape. **E**, **F** Module 2 **E** and the correlated enriched GO, KEGG pathways and Reactome Gene Sets **F** were analyzed with Cytoscape

The STRING database was used to construct the PPI network. DEGs between primary and lung metastatic CRC patients were uploaded to the STRING website to analyze the interaction relationships of those proteins. The top 10 hub genes, including 8 upregulated genes (*CLU*, *SFTPD*, *SFTPB*, *SFTPC*, *CCL18*, *SPP1*, *APOE* and *BGN*) and 2 downregulated genes (*MMP3* and *CXCL5),* were identified according to the highest degrees of connectivity using cytoHubba the plug-in in Cytoscape (Fig. 3A, B). In addition, module analysis was conducted by Molecular Complex Detection (MCODE) plug-in in Cytoscape and the top two significant modules are displayed in Fig. 3C, E. The analyses of GO function, KEGG pathways and Reactome Gene Sets indicated that these two modules were principally involved in positive regulation of ERK1/2 cascade, multicellular organismal process, extracellular matrix organization, surfactant metabolism and viral protein interaction with cytokines and cytokine receptors (Fig. 3D, F).

### Verification of hub gene expression between primary and metastatic CRC in the GEO database

**Fig. 4:**
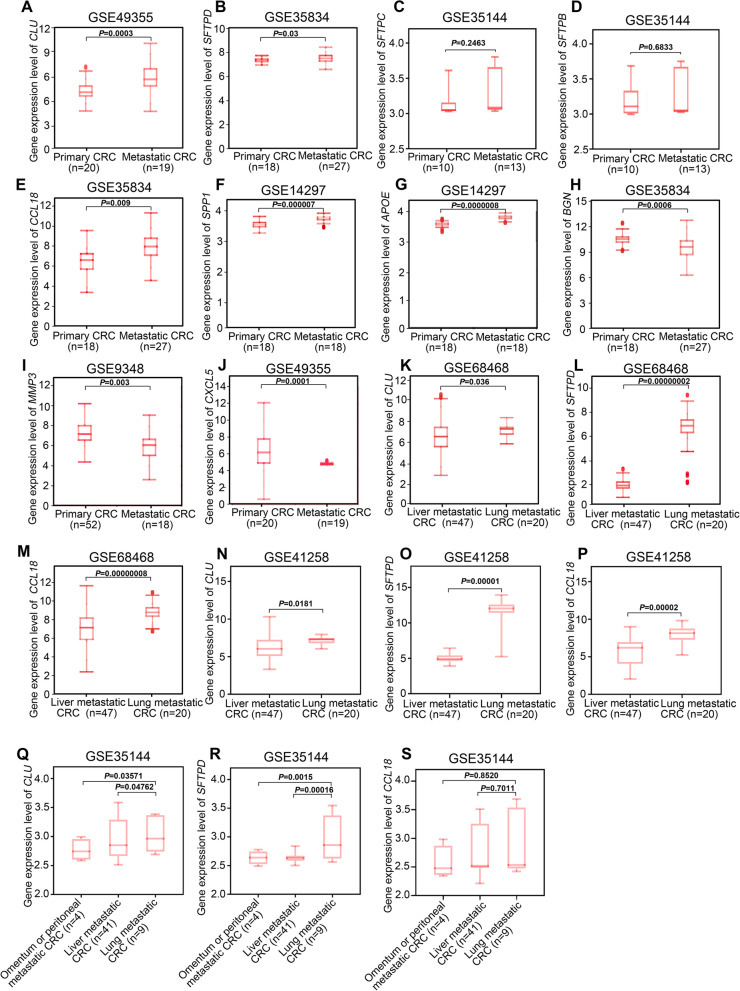
Expression levels of hub genes in colorectal cancer (CRC) patients from the GEO database. **A**-**J** The mRNA expression levels of the top 10 hub DEGs in primary and metastatic (including liver, lung, omentum or peritoneal) CRC were obtained from GEO datasets using Human Cancer Metastasis Database (HCMDB). **K**-**M** The transcriptional levels of *CLU*, *SFTPD* and *CCL18* in liver and lung metastatic CRC in GSE68468. **N**-**P** The transcriptional levels of CLU, SFTPD and CCL18 in liver and lung metastatic CRC in GSE41258. **Q**-**S** The transcriptional levels of CLU, SFTPD and CCL18 in liver, lung, omentum or peritoneal metastatic CRC in GSE35144. The data in **A**-**S** were normally distributed, and Student’s t test was used for results in **A**-**P**, and one-way ANOVA with post hoc intergroup comparison was used for results in **Q**-**S**. A *P* value < 0.05 was considered significant

To evaluate the above results from bioinformatic analysis, we further examined the transcriptional levels of hub genes in other GEO datasets. Consistent with results of GSE68468 and GSE41258, the mRNA levels of *CLU*, *SFTPD*, *CCL18, SPP1*, *APOE* and *BGN* were found to be significantly increased (Fig 4A, B AND E-H), while *MMP3* and *CXCL5* were notably decreased in metastatic CRC compared with primary CRC (Fig. 4I, J). However*,* no significant differences of transcriptional levels of *SFTPC* and *SFTPB* were observed between primary and metastatic CRC (Fig. 4C, D). Thus, we mainly focused on the 8 hub genes (*CLU*, *SFTPD*, *CCL18, SPP1* *,* *APOE*, *BGN MMP3* and *CXCL5*) except *SFTPC* and *SFTPB* in the following study.

Next, we investigated whether these eight hub genes were associated with organic tropism metastasis in CRC. The results in GSE68468, GSE41258 revealed that the expression levels of *CLU*, *SFTPD* and *CCL18* were particularly enhanced in CRC lung metastases compared with liver metastases (Fig. 4K-P). Additionally, similar expressional trends of those three genes were found in GSE35144 (Fig. 4Q-S). However, the expression levels of *SPP1*, *APOE*, *BGN, MMP3* and *CXCL5* were barely changed between lung metastatic CRC and liver, omentum or peritoneal metastatic CRC in those three CRC patient cohorts (Fig. S1). Collectively, these results suggest that *CLU*, *SFTPD* and *CCL18* might be important to drive the specific lung tropism metastasis in CRC.

### Prognostic analyses of hub genes in CRC

**Fig. 5:**
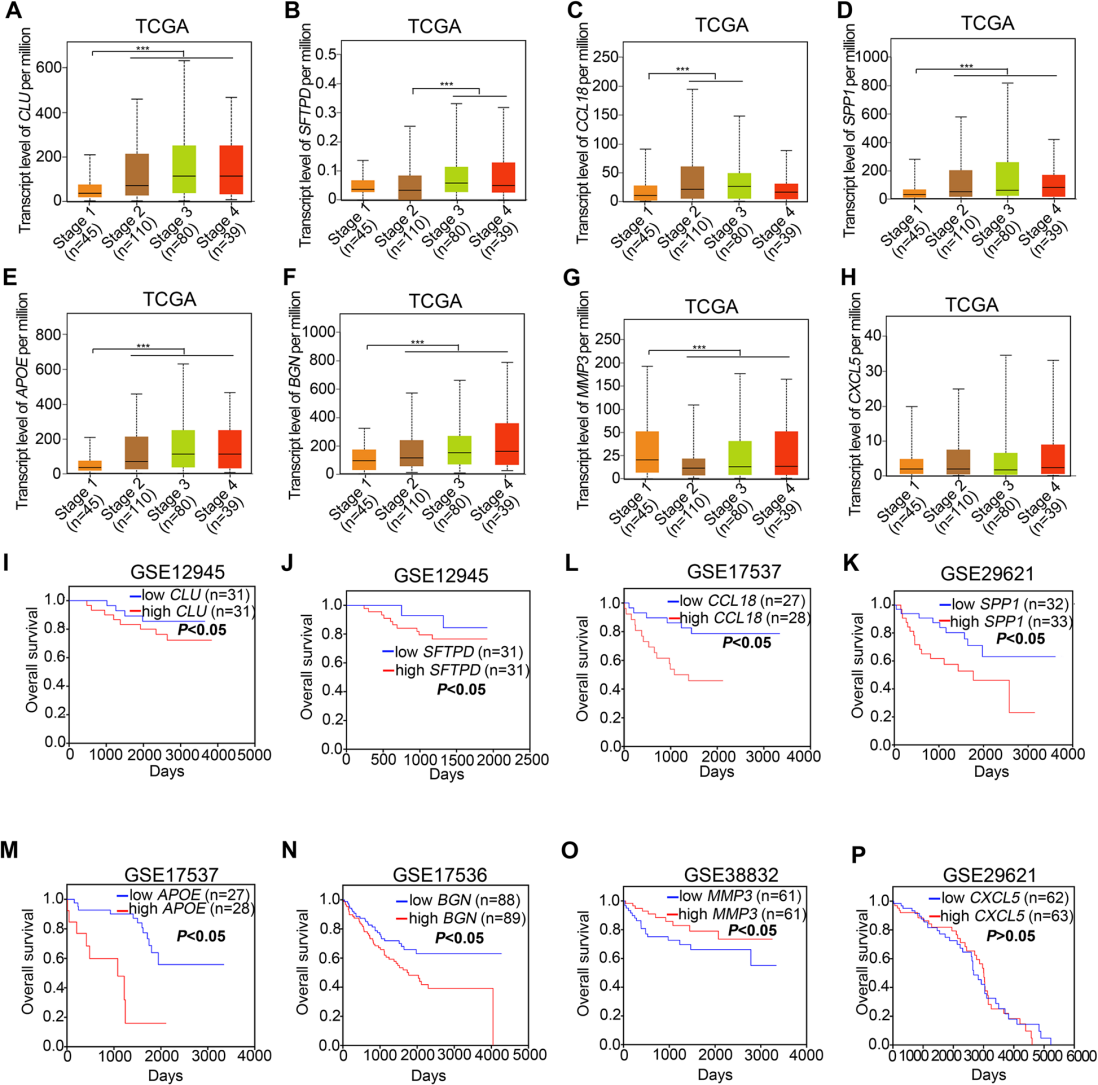
The prognostic value of hub genes in patients with colorectal cancer (CRC). **A**-**H** The mRNA expression of the hub DEGs in different CRC stages was from the TCGA database with the web tool UALCAN. Data were normally distributed and one-way ANOVA with post hoc intergroup comparison by Tukey's test was used. ***, *P* < 0.001. A *P* value < 0.05 was considered significant. **I**-**P** Relationships of hub DEG expression with overall survival in patients with CRC in the cohort of GEO databases are shown. Log-rank test. A *P* < 0.05 was considered significant

To explore the prognostic value of hub genes, we analyzed TCGA and GEO database on CRC patients and found that mRNA levels of *CLU*, *SFTPD*, *CCL18*, *SPP1*, *APOE* and *BGN* were upregulated at advanced CRC stages (Fig. 5A-F), while the expression of *MMP*3 was downregulated with the CRC stages (Fig. 5G). In addition, the expression level of *CXCL5* had no significant changes at different CRC stages (Fig. 5H). Furthermore, Kaplan–Meier survival analyses of the 8 hub genes in CRC patients were evaluated. The results revealed that high expression of *CLU*, *SFTPD*, *CCL18*, *SPP1*, *APOE* and *BGN* were positively associated with poor overall survival of CRC patients (Fig. 5I-N). However, high expression of *MMP3* was associated with longer overall survival (Fig. 5O). Additionally, similar to the expression status at different CRC stages, the transcriptional level of *CXCL5* was not significantly correlated with overall survival (Fig. 5P).

Collectively, integrative analysis of the results in Figs. 4 and 5 indicates that the expression levels of *CLU*, *SFTPD*, *CCL18*, *SPP1*, *APOE* and *BGN* in other GEO datasets and TCGA database showed a consistent increase in metastatic CRC compared with primary CRC in GSE68468 and GSE41258. And high expression of these genes was associated with advanced CRC stages and shorter overall survival. In addition, the expression levels of *MMP3* consistently decreased in CRC metastases compared with primary CRC tissues, and low expression of *MMP3* was also correlated with poor prognosis. The above findings imply that *CLU*, *SFTPD*, *CCL18*, *SPP1*, *APOE, BGN* and *MMP3* might play a critical role in CRC lung metastasis.

### Validation of the protein levels of hub genes in primary CRC and lung metastatic CRC

**Fig. 6:**
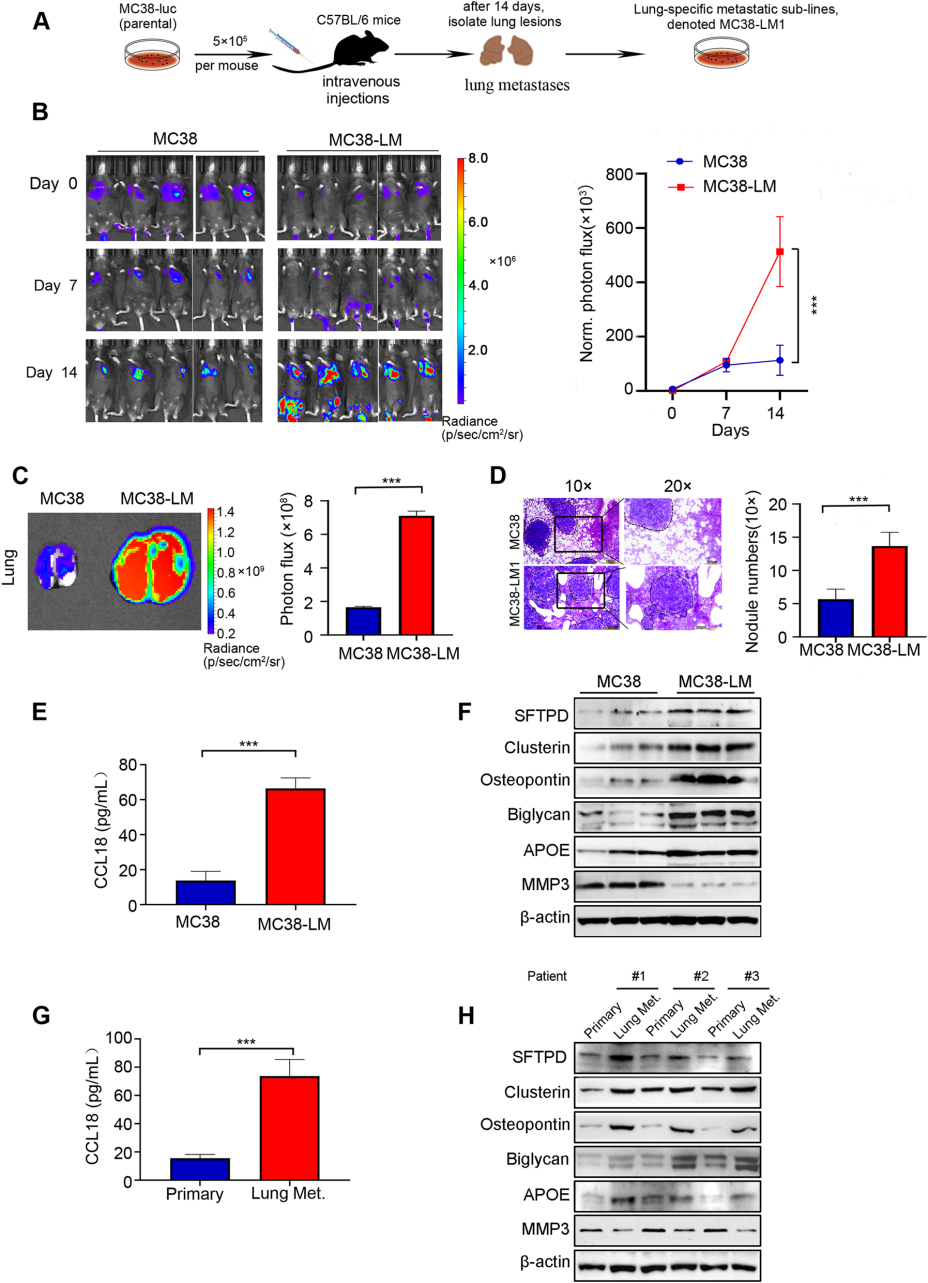
Expression levels of hub genes in primary and lung metastatic colorectal cancer (CRC) cells or tissues. **A** Graphic illustration of in vivo-selected highly lung metastatic CRC cells. MC38-Luc (5 × 10^5^) cells were intravenously injected into the C57BL/6 mice. After two weeks, a single lung metastatic nodule was isolated, dissociated and expanded to culture, yielding highly lung metastatic CRC cells, named MC38-Luc-LM. **B** Left, 5 × 10^5^ primary or lung metastatic MC38-Luc cells were intravenously injected into C57BL/6 mice and bioluminescence imaging (BLI) was captured weekly. Right, quantification of BLI photon flux at the lung zones of mice was conducted weekly. **C** Representative images and quantitative analysis of lung measured by BLI are shown on day14 after cell injection. **D** Representative images of H&E staining of lung and quantification of the lung metastatic nodules are presented. Scale bar: 200 µm (10 ×), 100 µm (20 ×). **E** The expression of CCL18 in the culture medium of primary and lung metastatic MC38-Luc cells detected by ELISA assay is shown. **F** The protein levels of SFTPD, Clusterin, Osteopontin, Biglycan, APOE and MMP3 in primary and lung metastatic MC38-Luc cells were examined by Western blotting assay. **G** The expression of CCL18 in the serum of primary and lung metastatic (Lung Met.) CRC patients detected by ELISA assay is shown. **H** The protein levels of SFTPD, Clusterin, Osteopontin, Biglycan, APOE and MMP3 in primary and lung metastatic (Lung Met.) patients with CRC were examined by Western blotting assay. For the results in (**B**-**E**) and (**G**), data were normally distributed and Student’s t test was used. ***, *P* < 0.001. Data are shown as means ± SD, *n* ≥ 3; A *P* < 0.05 was considered significant

Since the data in Fig. 5 suggested that the expression of *CXCL5* had no significant prognostic value in CRC patients, we mainly focused on the other 7 key hub genes (*CLU*, *SFTPD*, *CCL18*, *SPP1*, *APOE, BGN* and *MMP3*). To further validate protein levels of these key 7 hub genes in the progress of CRC lung metastasis, we established highly lung metastatic MC38-Luc cell sublines (named MC38-Luc-LM) through in vivo-selection as previously reported [19] (Fig. 6A). We next examined whether MC38-Luc-LM possessed highly lung metastasis capability. Primary MC38-Luc cells and lung metastatic MC38-Luc-LM cells were intravenously injected into C57/BL6 mice followed by weekly bioluminescence imaging (BLI) detection. No notable difference of BLI signal between the two groups after cell inoculation was observed on day 0 (Fig. 6B). Two weeks later, MC38-Luc-LM cells exhibited significantly enhanced lung metastatic competence compared to primary MC38-Luc cells, as evidenced by the elevated luciferase intensity in the lung zone, larger lung size, and increased number of lung metastatic nodules (Fig. 6B-D). In addition, no hepatic metastases were found at the end of experiment (Fig. S2).

We next measured expression levels of the above 7 hub genes in primary MC38-Luc and lung metastatic MC38-Luc (referred to MC38-Luc-LM) cells. Although these hub genes were expressed in normal lung, the mRNA levels of *Sftpd*, *Clu*, *Spp1*, *Bgn,* and *Apoe* were uniformly elevated, except that *Mmp3* level was lower, in lung metastatic MC38 cells than in primary MC38 cells (Fig. S3). The mRNA level of *CCL18* was not examined due to its unknown gene sequence in mice. In addition, the results of Western blotting revealed that the protein levels of SFTPD, Clusterin (encoded by *CLU*), Osteopontin (encoded by *SPP1*), Biglycan (encoded by *BGN*), APOE, and CCL18 were dramatically upregulated other than MMP3 downregulated in lung metastatic MC38 cells compared with primary MC38 cells (Fig. 6E, F and Fig. S4). Furthermore, similar results were observed in lung metastases of CRC patients compared with primary CRC (Fig. 6G, H).

### SFTPD contributes to malignant phenotypes of colorectal cancer

**Fig. 7:**
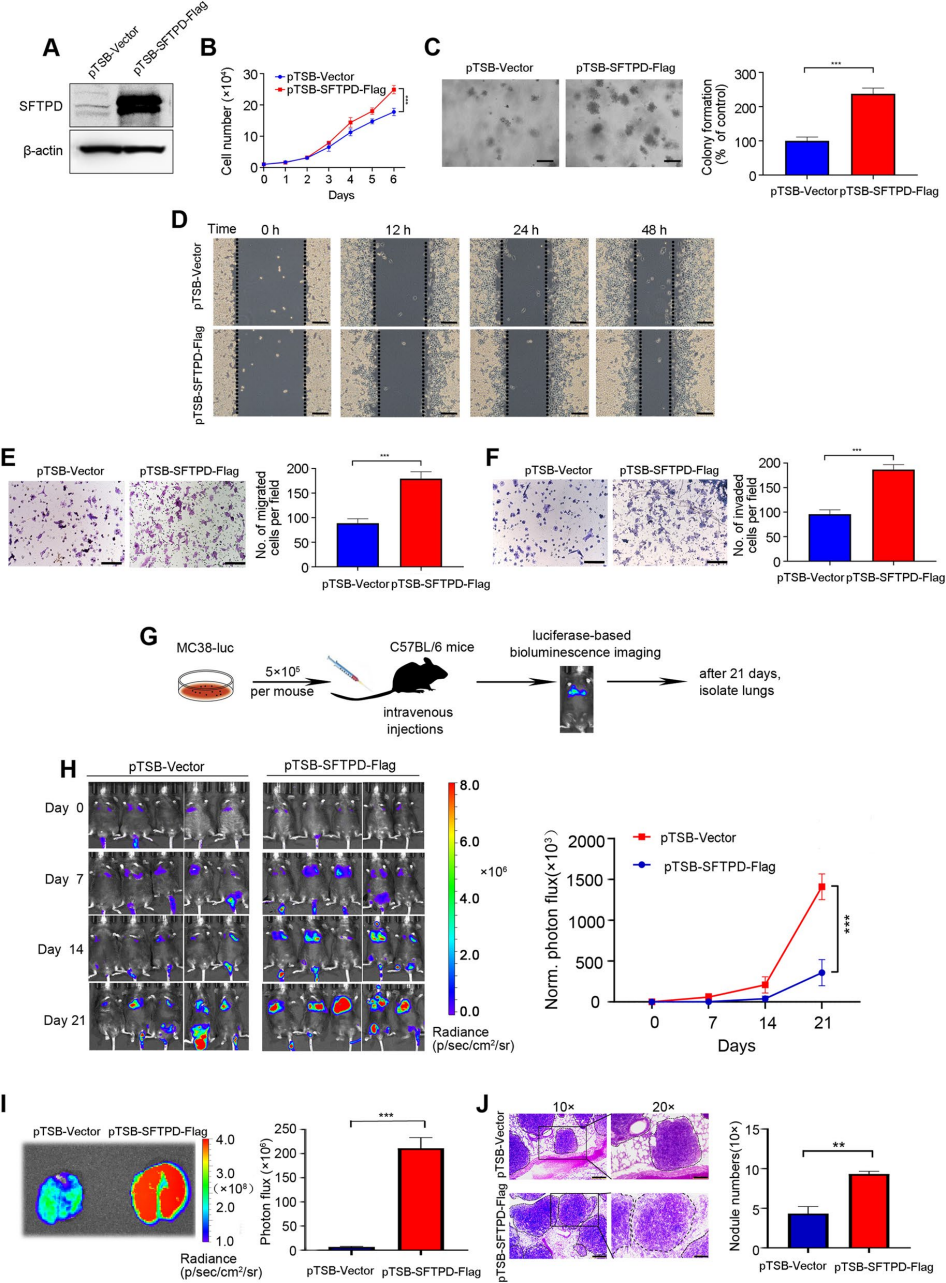
SFTPD promotes lung metastasis of colorectal cancer (CRC) cells. **A** The overexpression efficiency of SFTPD in MC38-Luc cells was examined by Western blotting. **B** Forced SFTPD expression facilitated proliferation in MC38-Luc cells as determined by trypan blue exclusion assay. **C** SFTPD overexpression enhanced the ability of colony formation in MC38-Luc cells as determined by the soft agar assay. **D** Representative images of the wound healing scratch assay from MC38-Luc cells stably expressing the vector or SFTPD cDNA construct are shown. Scale bar: 200 μm. **E** The representative images of migrated cells (left) and the responding quantitative data (right) in MC38-Luc cells stably expressing the vector or SFTPD cDNA construct are shown.** F** Representative images of invaded cells (left) and the corresponding quantitative data (right) in MC38-Luc cells stably expressing the vector or SFTPD cDNA construct are shown. **G** Graphic illustration of the lung metastasis mouse model of CRC. MC38-Luc cells (5 × 10^5^) stably expressing the vector or SFTPD cDNA construct were intravenously inoculated into the C57BL/6 mice. BLI was performed weekly for 3 weeks. **H** Representative photographs of mice by BLI weekly and quantification of BLI photon flux at lung zones of mice was conducted. **I** Representative images and quantitative analysis of the lung measured by BLI are shown on day 21 after cells injection.** J** Representative images of H&E staining of the lung and quantification of lung metastatic nodules are presented. Scale bar: 200 µm (10 ×), 100 µm (20 ×). For the results in (**B**-**C**), (**E**–**F**) and (**H**-**J**), data were normally distributed and Student’s* t* test was used**.** **, *P* < 0.01; ***, *P* < 0.001. Data are shown as means ± SD, n ≥ 3; A *P* < 0.05 was considered significant

To further confirm the accuracy and reliability of the above bioinformatics analysis, *SFTPD,* one of the three hub genes (*CLU*, *SFTPD* and *CCL18*) that was not only specifically boosted in lung metastatic CRC compared with liver metastases, but also significantly correlated to poor prognosis of CRC patients, was selected for subsequent functional experiment. MC38-Luc cells stably over-expressing SFTPD was constructed (Fig. 7A), and we found that SFTPD overexpression markedly enhanced cell proliferation and the clonogenicity compared with vector-expressing cells (Fig. 7B-C). In addition, the overexpression of SFTPD in MC38-Luc cells significantly increased the migration and invasion ability as assayed by wound healing assays and Transwell assays (Fig. 7D-F).

To verify whether SFTPD efficiently promotes lung metastasis in CRC, we intravenously inoculated MC38-Luc cells stably expressing vector or SFTPD into C57BL/6 mice (Fig. 7G). Overexpression of SFTPD led to a substantial increase in BLI signals in the lung zones of mice (Fig. 7H). Moreover, the intensity of BLI signals and the size of dissected lungs were obviously increased in mice injected with SFTPD-overexpressing cells compared to vector control cells (Fig. 7I). Importantly, H&E staining displayed more metastatic nodules in lungs of mice with SFTPD-overexpressing CRC cells (Fig. 7J). Together, these results indicate that SFTPD significantly promotes lung metastasis of CRC.

## Discussion

Understanding the underlying molecular mechanisms of CRC lung metastasis would greatly benefit diagnosis, management and prognosis evaluation. In the present study, we identified 57 highly expressed DEGs and 18 poorly expressed DEGs between primary CRC samples and lung metastatic CRC samples by analyzing microarrays in the GEO database. The enrichment of these deregulated genes revealed that core pathways and hub genes could lead to new insights into CRC lung metastasis.

As suggested by GO analysis, the dysregulated genes were mainly enriched in inflammatory response, chemotaxis, chemokine activity, immune response, immunoglobulin receptor binding, antigen binding, cell adhesion and positive regulation of ERK1/2 cascade. This is plausible since inflammatory responses are important in the progression of cancer, including tumour initiation and metastasis. As main inflammatory mediators, chemokine activity, chemotaxis or aberrant immune reactions are critical tumourigenic signals of CRC [20, 21]. Cell adhesion molecules play a significant role in cell permeability, polarity and migration, which are the vital steps in CRC progression and metastasis [22]. In addition, it has been reported that the dysfunction of ERK/MAPK pathway is a crucial trigger for the progression of most cancers [23]. Moreover, the DEGs were also found to be enriched in the formation of ECM, extracellular exosomes, extracellular space and so on, indicating that the interaction with the extracellular environment could be triggered during the CRC lung metastatic process.

The KEGG and Reactome Gene Sets analyses of DEGs and module analyses of the PPI network suggested that surfactant metabolism, phagosomes, cell–cell communication, and ECM organization may be involved in CRC lung metastasis, except for cell adhesion molecules and chemokine signaling pathways, which have already been found in GO enrichment. To date, no direct evidence of the role of surfactant metabolism in CRC lung metastases has been presented. Several studies reported the interaction between cancer metastasis and surfactant metabolism. It has been demonstrated that pulmonary and extra-pulmonary existence of surfactant proteins play important roles in film stabilization, viral defense and modulation of immune responses [24]. In the current study, the expression of SFTPB, SFTPC, SFTPD, and ABCA3, which are involved in the production, function, and metabolism of surfactant [25], was shown to be highly expressed in CRC lung metastases, suggesting that they may promote CRC lung metastasis. Phagosomes are dynamic organelles generated within cells by the uptake of particles larger than 0.5 μm, which are essential for pathogen eradication and antigen presentation in the process of innate and adaptive immunity [26]. Emerging evidence highlights the effect of immune microenvironment on colorectal metastasis [27]. This implies that DEGs associated with phagosomes formation and maturation might participate in CRC lung metastasis by influencing immunity.

Cell–cell communication is crucial for several biological events, including cell fate determination, proliferation, migration, and homeostasis. It has been well recognized that cell–cell communication between tumour microenvironments (e.g., stromal fibroblasts, epithelial cells, and multiple immune cell-types) and cancer cells drives CRC metastasis [28, 29]. ECM consists of various molecules, such as laminin, collagen, elastin and fibronectin, and plays a central role in tumour initiation, progression, and metastasis. Cross-talk between the ECM and CRC metastasis has been well clarified in the previous report [30]. Dysregulated ECM-related proteins induce both biochemical and biomechanical changes to promote cancer metastasis [18]. Herein, the upregulated expression of MGP, Biglycan, LTBP2 and PRELP may facilitate the interactions between CRC cells and ECM, and therefore promote cellular survival and colonization in CRC lung metastases. The enriched pathways modulated by DEGs in this study could provide some rationales for developing novel therapeutic targets in the treatment of CRC.

Of importance, the top 10 hub genes were identified in CRC lung metastases, including 8 upregulated genes and 2 downregulated genes. We validated the transcriptional expression of the hub genes in numerous primary and metastatic CRC cases in the GEO database. The expression of these hub genes was in accordance with data obtained through bioinformatics analysis in GSE41258 and GSE68468. The prognostic values of these hub genes were further analyzed in the TCGA and GEO database. High expression levels of *CLU*, *SFTPD*, *CCL18*, *SPP1*, *APOE* and *BGN* were positively associated with poor overall survival of CRC patients and low expression of *MMP3* was associated with longer overall survival. Therefore, we hypothesized that *CLU, SFTPD, CCL18, SPP1, APOE, BGN* and *MMP3* might be candidate biomarkers in CRC lung metastasis. To test this hypothesis, we examined protein levels of the seven genes in primary and highly lung metastatic MC38 cells, and paired CRC primary and lung metastatic tissues. Consistently, the protein expression levels of Clusterin*,* SFTPD, CCL18, Osteopontin*,* APOE, and Biglycan were significantly higher, and MMP3 was lower in lung metastatic CRC cells or tissues than in primary CRC cells or tissues.

Among seven core genes, the expression levels of *SPP1, APOE,* and *BGN* were found to be upregulated, while *MMP3* was downregulated in CRC lung metastases compared with primary CRC. Indeed, several studies have demonstrated that *SPP1, APOE,* and *BGN* could be involved in the CRC malignant phenotype [31–33]. *SPP1,* encoding by Osteopontin, is an ECM protein which is reported to be overexpressed in a variety of malignancies such as ovarian cancer, breast cancer and CRC [31, 34, 35]. Osteopontin has been reported to boost the abilities of cell survival, migration, and angiogenesis to drive tumourgenesis and metastasis in CRC [31]. *APOE,* encoding Apolipoprotein E (APOE), is critical for lipoprotein metabolism [36]. Recent studies have demonstrated that APOE also contributes to DNA synthesis, cell proliferation, angiogenesis, and metastasis to facilitate tumorigenesis and progression [37]. Similar to previous reports that APOE was increased in CRC liver metastases [32], we found that APOE was elevated in CRC lung metastases and was positively associated with advanced stages and poor overall survival in CRC. *BGN* encodes Biglycan, which is a widely expressed ECM protein that provides stability and organization in tissues by interacting with other ECM proteins such as collagen and elastin [38]. Biglycan has been reported to trigger the activation of several pathways involved in tumorigenesis by orchestrating growth factors/cytokines and cell surface receptors [39]. In CRC, high level of Biglycan has been linked with metastatic progression, poor prognosis [33]. MMP3, also commonly known as matrix metallopeptidase 3, is encoded by *MMP3* and belongs to a group of zinc-dependent proteolytic enzymes. Moran et al. reported that MMP3 expression was lower in CRC patients with high microsatellite instability (MSI) when compared with low or null MSI [40]. However, compelling evidence has shown that MMP3 promotes cancer invasion and metastasis by cleaving E-cadherin and disrupting its interaction with β-catenin [41, 42]. Some studies reporting that MMP3 exhibits anti-tumour activities depending in a substrate-depend manners [43, 44]. For instance, MMP3-mediated cleavage of IGF-BP3 and IGF-BP5 inhibits tumorigenesis in breast cancer [44]. Herein, MMP3 was shown to hamper CRC lung metastasis with unknown substrates, which needs further investigation.

Since retrospective clinical data reveal that 24.5% of metastatic CRC patients first develop lung metastases and lung metastases account for 32.9% of all metastatic CRCs [4], we focused on CRC lung metastasis in the present study. Here, we found that the expression levels of *CLU*, *CCL18,* and *SFTPD* were especially upregulated in CRC lung metastases instead of other metastases, and were positively associated with poor prognosis of CRC patients. Clusterin encoded by *CLU*, functions as a stress-activated molecular chaperone that is highly expressed in aggressive cancers by modulating different signaling networks [45]. It plays important roles in the regulation of protein homeostasis, pro-survival signaling and transcriptional networks [46]. Studies have demonstrated that high Clusterin expression is associated with a shorter survival time and that could be the biomarker for CRC patients [47, 48]. Therefore, targeting Clusterin might be a promising approach for the management of CRC. *CCL18* encodes CC chemokine ligand 18 (CCL18), which is mainly expressed by macrophages and dendritic cells. CCL18 has been implicated in the stimulation of angiogenesis as well as cancer cell migration, invasion, and epithelial-to-mesenchymal transition. Recent studies have demonstrated that high expression of CCL18 in CRC patients is correlated with advanced tumour staging and liver metastasis [49, 50], which is similar to our findings in lung metastasis of CRC. Surfactant protein D (also known as SFTPD or SP-D), encoded by the *SFTPD* gene, is a collagenous glycoprotein that resides in the lungs and extra-pulmonary tissues [51]. To date, only one study has reported that SFTPD is negatively associated with pulmonary metastases in CRC [52]. However, our in vitro and in vivo results showed that SFTPD promotes cellular proliferation, migration, and invasion and further enhanced CRC cell lung metastasis*.* This inconsistent finding could be due to different cellular contexts and animal models.

In the current study, we highlighted that *CLU*, *SFTPD,* and *CCL18* might serve as potential targets for the treatment of CRC lung metastasis. The effect of SFTPD on CRC lung metastasis was investigated through in vitro and in vivo experiments. Further investigation is warranted, especially to determine the precise mechanisms underlying the effect of these hub genes on CRC.

## Conclusion

In summary, our bioinformatics analysis identified the DEGs and hub genes implicated in lung metastatic CRC, which may play critical roles in regulating CRC lung metastasis. A total of 75 DEGs and 10 hub genes were defined, and the enrichment analysis suggests that surfactant metabolism might play a dominant role in CRC lung metastasis. The 7 core hub genes, including *CLU*, *SFTPD*, *CCL18*, *SPP1*, *APOE, BGN* and *MMP3,* were significantly correlated with the advanced CRC stages and poor prognosis. Importantly, *CLU, SFTPD and CCL18* might positively be correlated with specific lung tropism metastasis in CRC and represented as potential targets for the prevention and treatment of patients with CRC lung metastasis. Of note, our study demonstrated that SFTPD was critical to drive CRC lung metastasis. These findings may contribute to a profound understanding of CRC lung metastasis. Further studies are warranted to validate the results of these findings.

## Supplementary Information

**.Additional file 1: Fig. S1. **Expression levels of hub genes in metastatic colorectal cancer (CRC) patients in the GEO database.

**Additional file 2: Fig. S2.** No liver metastatic nodules were found in the lung metastatic mouse model.

**Additional file 3: Fig. S3.** qRT-PCR analyses of hub genes expression in mouse normal lung tissues, primary MC38 cells and lung metastatic MC38 cells.

**Additional file 4: Fig. S4.** Quantitative analysis of hub genes in primary MC38 cells and lung metastatic MC38 cells.

**Additional file 5: Table S1. **Mouse primers for qRT-PCR.

**Additional file 6: Table S2. **The fifty-seven upregulated DEGs in the GSE41258 and GSE68468 dataset.

**Additional file 7: Table S3. **The eighteen downregulated DEGs in the GSE41258 and GSE68468 dataset.

**Additional file 8: Original blots of Fig. 6.**

**Additional file 9: Origi nal bl ots of Fig. 7.**

## Data Availability

The datasets generated and analyzed during the current study are available in the TCGA GDC repository, (https://portal.gdc.cancer.gov), GEO repository, (https://www.ncbi.nlm.nih.gov/geo/), Human Cancer Metastasis Database, (http://hcmdb.i-sanger.com/).

## Abbreviations

ATCC: American Type Culture Collection
BLI: Bioluminescence imaging
BP: Biological processes
CC: Cell component
CRC: Colorectal cancer
DEGs: Differentially expressed genes
ELISA: Enzyme-linked immunosorbent assay
ECM: Extracellular matrix
GEO: Gene expression omnibus
GO: Gene Ontology
HCMDB: Human Cancer Metastasis Database
KEGG: Kyoto encyclopedia of genes and genomes
MCODE: Molecular complex detection
MF: Molecular function
MSI: Microsatellite instability
PPI: Protein–protein interaction
STRING: Search tool retrieval of interacting genes
TCGA: The Cancer Genome Atlas
