# Supplementary material for: Identification of hub genes and pathways in lung metastatic colorectal cancer
Source: BMC Cancer. 2023 Apr 6;23:323. doi: 10.1186/s12885-023-10792-8 (PMC10080892; doi:10.1186/s12885-023-10792-8)
Supplement: Supplementary file 1 — .Additional file 1: Fig. S1. Expression levels of hub genes in metastatic colorectal cancer (CRC) patients in the GEO database. [file 12885_2023_10792_MOESM1_ESM.pdf]

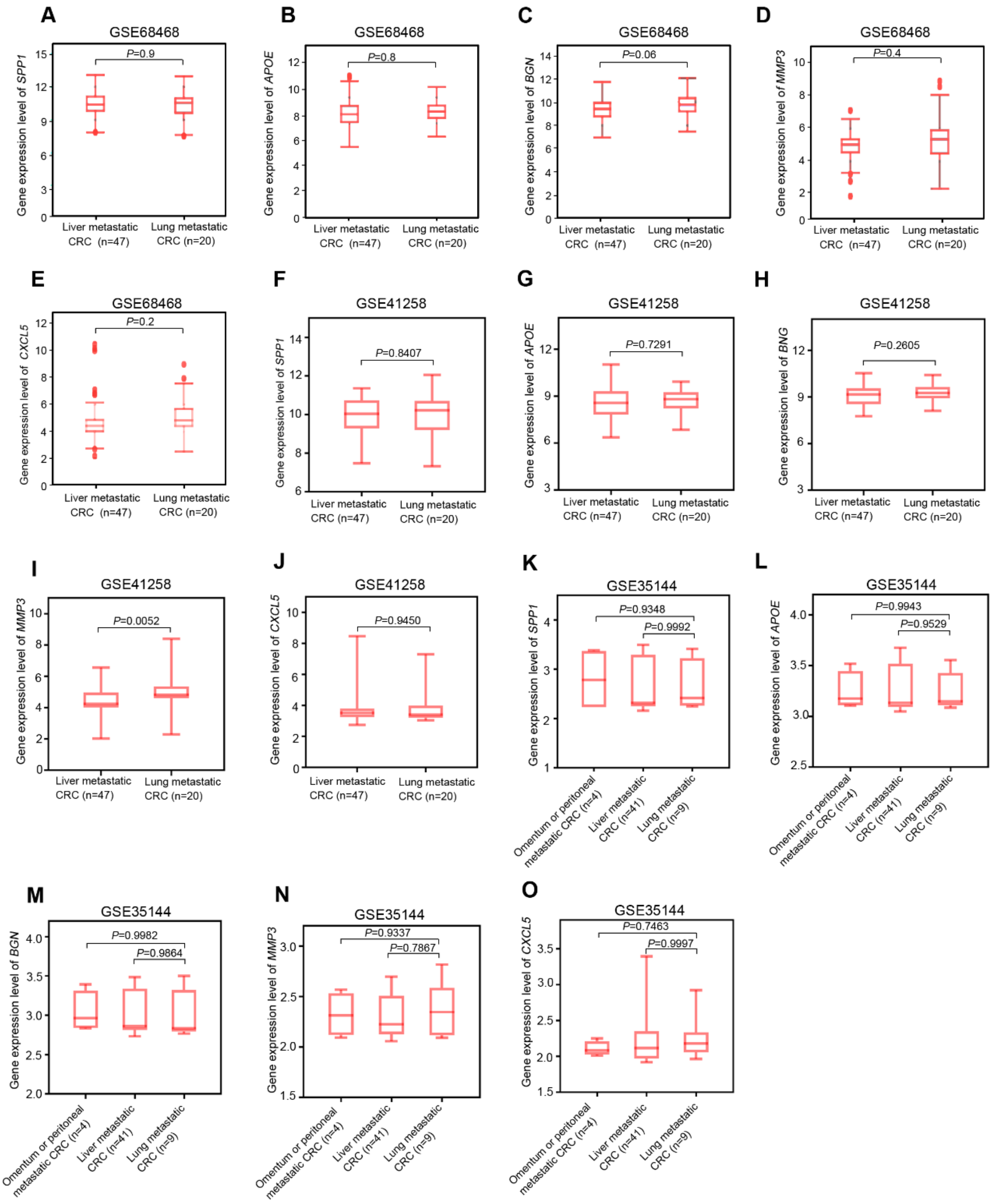

Fig. S1 Expression levels of hub genes in metastatic colorectal cancer (CRC) patients in the GEO database. (A-E) The transcriptional levels of *SPP1*, *APOE*, *BGN, MMP3* and *CXCL5* in the liver and lung metastatic CRC in GSE68468. **(F-J)** The transcriptional levels of *SPP1*, *APOE*, *BGN, MMP3* and *CXCL5* in the liver and lung metastatic CRC in GSE41258. **(K-O)** The transcriptional levels of *SPP1*, *APOE*,

BGN, MMP3 and *CXCL5* in the liver, lung, omentum or peritoneal metastatic CRC in GSE35144. The data in (A-O) were normally distributed, and Student's *t* test was used for results in (A-J), and one-way ANOVA with *post hoc* intergroup comparison was used for results in (K-O). A P value < 0.05 was considered significant.
