# Supplementary material for: Identification of hub genes and pathways in lung metastatic colorectal cancer
Source: BMC Cancer. 2023 Apr 6;23:323. doi: 10.1186/s12885-023-10792-8 (PMC10080892; doi:10.1186/s12885-023-10792-8)
Supplement: Supplementary file 2 — Additional file 2: Fig. S2. No liver metastatic nodules were found in the lung metastatic mouse model. [file 12885_2023_10792_MOESM2_ESM.pdf]

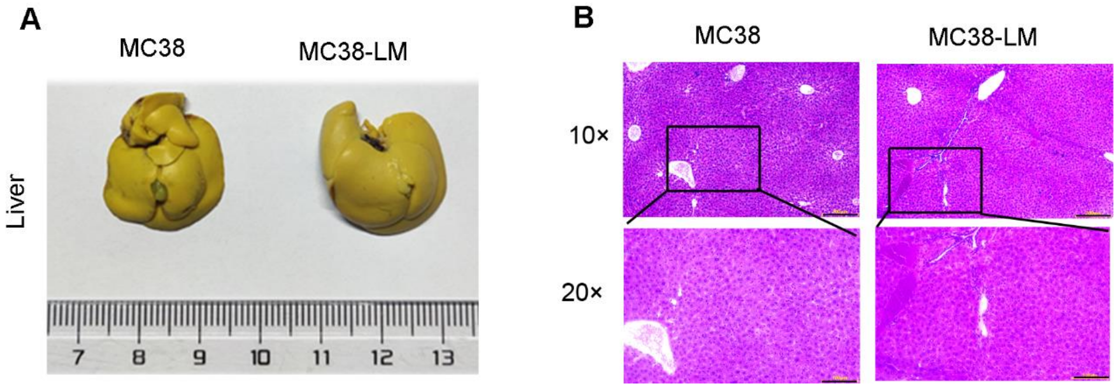

Fig. S2. No liver metastatic nodules were found in the lung metastatic mouse model. (A) Representative images of liver after Bouin's fixation for one week. **(B)** Representative images of H&E staining of liver section. Scale bar: 200 μm (10×), 100 μm (20×).
