# Supplementary material for: Identification of hub genes and pathways in lung metastatic colorectal cancer
Source: BMC Cancer. 2023 Apr 6;23:323. doi: 10.1186/s12885-023-10792-8 (PMC10080892; doi:10.1186/s12885-023-10792-8)
Supplement: Supplementary file 3 — Additional file 3: Fig. S3. qRT-PCR analyses of hub genes expression in mouse normal lung tissues, primary MC38 cells and lung metastatic MC38 cells. [file 12885_2023_10792_MOESM3_ESM.pdf]

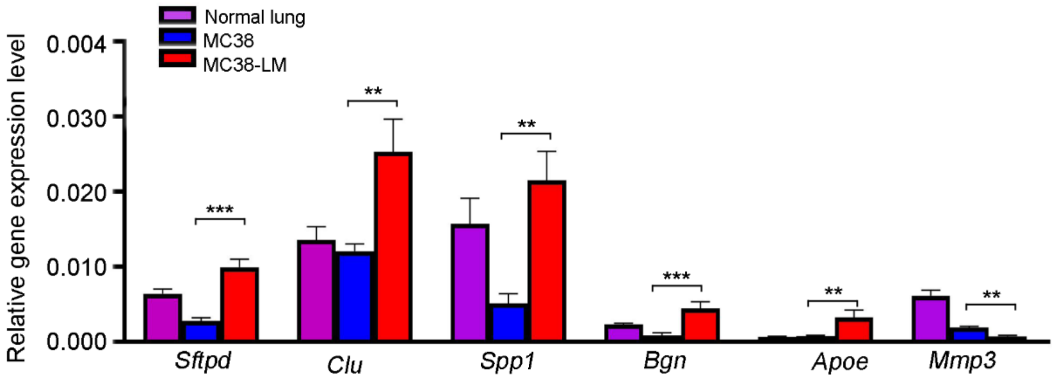

Fig. S3. qRT-PCR analyses of hub genes expression in mouse normal lung tissues, primary MC38 cells and lung metastatic MC38 cells. (A) The mRNA levels of *Sftpd*, *Clu*, *Spp1*, *Apoe*, *Bgn* and *Mmp3* in mouse normal lung tissues, primary MC38 cells and lung metastatic MC38 cells are shown. The data were normally distributed, and one-way ANOVA with *post hoc* intergroup comparison was used. **, $P < 0.01$; ***, $P < 0.001$. Data are shown as means ± SD, n ≥ 3; A $P < 0.05$ was considered significant.
