# Supplementary material for: Identification of hub genes and pathways in lung metastatic colorectal cancer
Source: BMC Cancer. 2023 Apr 6;23:323. doi: 10.1186/s12885-023-10792-8 (PMC10080892; doi:10.1186/s12885-023-10792-8)
Supplement: Supplementary file 4 — Additional file 4: Fig. S4. Quantitative analysis of hub genes in primary MC38 cells and lung metastatic MC38 cells. [file 12885_2023_10792_MOESM4_ESM.pdf]

Table S1. Mouse primers for qRT-PCR

Gene	Forward	Reverse
Sftpd	5'-TCTCCCACTATCAGAAAGCTGC-3'	5'-CTCAGTAGCAGAACGTGGGG-3'
Clu	5'-GGGGTGTACTTGAGCAGAGC-3'	5'-TCCTTGGAATCTGGAGTCCGG-3'
Spp1	5'-AGGAAACCAGCCAAGGACTAAC-3'	5'-GCTTCTTCTCCTCTGAGCTGC-3'
Bgn	5'-GCGGAAGCTGCAAAAACTCT-3'	5'-ACCTGTGTCCTTTATGCCTGA-3'
Apoe	5'-GTCCCAGCCTTGGAATGGAA-3'	5'-GCTGGTCAGCCTAAACTGGAA-3'
Mmp3	5'-CAGTCCCTCTATGGAACTCCC-3'	5'-AGGGTGCTGACTGCATCAAA-3'
Gapdh	5'-ACCCTTAAGAGGGATGCTGC-3'	5'-CCCAATACGGCCAAATCCGT-3'
