# Supplementary material for: Identification of hub genes and pathways in lung metastatic colorectal cancer
Source: BMC Cancer. 2023 Apr 6;23:323. doi: 10.1186/s12885-023-10792-8 (PMC10080892; doi:10.1186/s12885-023-10792-8)
Supplement: Supplementary file 5 — Additional file 5: Table S1. Mouse primers for qRT-PCR. [file 12885_2023_10792_MOESM5_ESM.pdf]

Table S2. The fifty-seven upregulated DEGs in the GSE41258 and GSE68468 dataset

Gene Symbol	Gene ID	Log_2 FC in GSE41258	Log_2 FC in GSE68468	Adj *P* Value in GSE41258	Adj *P* Value in GSE68468
SFTPC	6440	8.3322819	5.9318462	1.36E-136	3.59E-71
SFTPB	6439	6.479101	6.124719	1.37E-84	1.54E-66
SFTPD	6441	6.0460797	4.8391114	3.35E-65	1.77E-58
SFTPA2	729238	5.7916673	6.19483	1.57E-64	3.69E-68
SCGB1A1	7356	2.4725687	3.3677696	2.23E-15	1.10E-24
LTF	4057	2.2917249	4.1286066	1.29E-17	4.94E-27
CCL18	6362	1.99271	2.2897545	0.000103	2.70E-04
IGHM	3507	1.9294555	2.1339972	0.000159	7.28E-05
CYP1B1	1545	1.9088015	2.2351386	0.000000286	3.94E-06
IGLC1	3537	1.887292	1.940792	0.0165	2.18E-02
C7	730	1.870994	2.3569682	0.000000366	8.48E-07
OR7A10	390892	1.8120841	1.3723377	0.0000378	1.05E-05
IGLL1	3543	1.8020492	1.0546332	0.000541	2.68E-04
ABCA3	21	1.7859522	1.5321818	1.71E-10	1.03E-06
SLC34A2	10568	1.7747287	2.8237262	2.42E-33	2.52E-35
ITGBL1	9358	1.765783	2.338059	8.91E-05	7.94E-05
ADH1B	125	1.724882	1.8651	0.000554	1.10E-03
NKX2-1	7080	1.710947	2.045118	5.07E-06	1.86E-06
IGHD	3495	1.695941	3.129595	2.16E-08	2.00E-09
IGLV1-44	28823	1.652244	1.937716	0.0314	1.40E-03
MGP	4256	1.636953	1.872567	1.75E-05	3.95E-04
APOE	348	1.607058	1.497875	0.000177	3.27E-03
MARCO	8685	1.559874	2.677586	2.39E-06	7.13E-11
SPP1	6696	1.492127	1.61012	0.00151	5.25E-03
CLU	1191	1.487431	1.577097	0.00116	6.74E-03
CCL19	6363	1.467586	1.721906	1.02E-05	5.94E-06
APOC1	341	1.451887	1.973934	0.000411	1.44E-04
LOC100653057	100653057	1.447004	2.004486	5.27E-06	4.24E-05
IGLL3P	91353	1.436033	1.811463	0.00226	9.56E-05
FMO2	2327	1.393507	1.92863	8.09E-35	1.55E-34
GUSBP11	91316	1.311374	1.309558	0.00646	1.21E-03
POU2AF1	5450	1.302847	1.392807	0.00114	1.14E-03
EGFL6	25975	1.295546	1.345563	3.11E-14	4.65E-12
CADM1	23705	1.278576	1.173003	5.68E-10	9.24E-05
BMS1P20	96610	1.276755	1.696582	0.0101	3.10E-04
IGHV4-31	28396	1.267629	1.405921	0.0401	1.06E-02
HBA2	3040	1.257768	1.396487	0.000414	1.96E-02
PTGIS	5740	1.193255	1.406424	0.000233	4.19E-04
CYAT1	100290481	1.17631	1.862045	0.00932	5.49E-03
LTBP2	4053	1.175268	1.222965	3.71E-10	1.01E-06

RPS11	6205	1.153035	2.247998	2.04E-06	1.34E-08
CYP4B1	1580	1.145366	3.421862	3.58E-12	4.62E-19
PRELP	5549	1.145163	1.95971	1.07E-07	2.57E-05
MIR8071-2	102466889	1.144333	1.69677	0.00571	1.02E-02
BGN	633	1.125545	1.557654	2.34E-05	5.46E-05
C4BPA	722	1.103133	1.649178	2.35E-08	3.42E-11
IGHV3-23	28442	1.079822	1.648242	0.0394	2.58E-02
ENPP2	5168	1.073004	1.19066	1.22E-06	1.73E-04
CLDN5	7122	1.069629	1.454305	1.51E-06	8.59E-05
IGLJ3	28831	1.061169	1.698304	0.0245	1.29E-02
PTP4A3	11156	1.057674	1.339006	1.43E-06	3.98E-06
BNIP3	664	1.053185	1.356462	0.000809	3.53E-03
MYH10	4628	1.025988	1.488319	8.68E-07	1.48E-04
RPL37A	6168	1.018398	1.154699	7.97E-09	5.73E-08
SLIT2	9353	1.012822	1.130593	1.29E-10	3.15E-07
SERPINB3	6317	1.007778	1.449198	0.0029	4.17E-04
CNGB1	1258	1.002436	1.053638	7.31E-11	3.00E-04
