# Supplementary material for: Identification of hub genes and pathways in lung metastatic colorectal cancer
Source: BMC Cancer. 2023 Apr 6;23:323. doi: 10.1186/s12885-023-10792-8 (PMC10080892; doi:10.1186/s12885-023-10792-8)
Supplement: Supplementary file 6 — Additional file 6: Table S2. The fifty-seven upregulated DEGs in the GSE41258 and GSE68468 dataset. [file 12885_2023_10792_MOESM6_ESM.pdf]

Table S3. The eighteen downregulated DEGs in the GSE41258 and GSE68468 dataset

Gene Symbol	Gene ID	Log_2 FC in GSE41258	Log_2 FC in GSE68468	Adj *P* Value in GSE41258	Adj *P* Value in GSE68468
MAB21L2	10586	-2.5946	-2.17959	7.00E-16	1.34E-11
SPINK4	27290	-2.47337	-1.77526	0.000208	4.38E-03
MMP3	4314	-2.23088	-2.44694	1.34E-08	3.36E-08
MUC2	4583	-1.79232	-1.61319	0.00453	2.23E-02
PLA2G2A	5320	-1.77058	-1.55786	0.00415	8.60E-03
ZG16	653808	-1.66785	-1.52806	0.00478	3.30E-02
CXCL14	9547	-1.60283	-1.28666	0.000317	4.96E-02
NMU	10874	-1.45997	-2.03069	0.000296	1.06E-03
PCK1	5105	-1.40534	-1.17076	0.0113	5.76E-02
FUT6	2528	-1.37873	-1.42724	0.000207	1.02E-02
ACTG2	72	-1.29196	-1.23175	0.00191	6.60E-03
ADAMDEC1	27299	-1.25617	-1.0073	0.000316	1.99E-02
BAX	581	-1.24235	-1.58579	6.89E-07	6.11E-10
GREM1	26585	-1.20482	-1.18975	0.000545	0.00259
CXCL5	6374	-1.17421	-1.4844	0.0379	5.76E-02
DES	1674	-1.08758	-1.77719	0.0143	4.17E-03
ABHD2	11057	-1.04661	-1.29764	0.00104	0.000564
CLIC4	25932	-1.04147	-1.17762	0.0000102	0.00013
