# Supplementary figures and images for: Identification of hub genes and pathways in lung metastatic colorectal cancer
Source: BMC Cancer. 2023 Apr 6;23:323. doi: 10.1186/s12885-023-10792-8 (PMC10080892; doi:10.1186/s12885-023-10792-8)

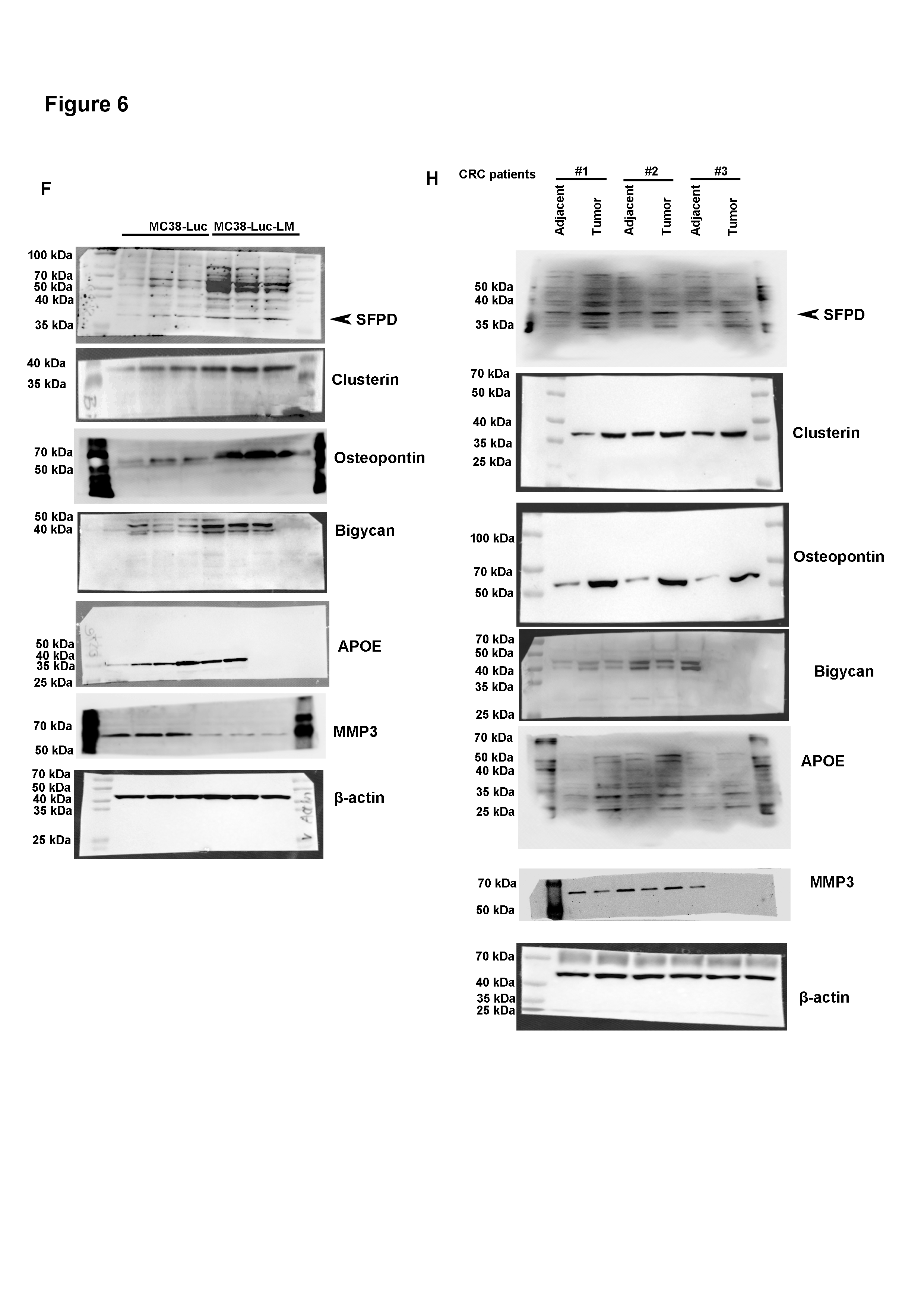

Supplement: Supplementary file 7 — Additional file 7: Table S3. The eighteen downregulated DEGs in the GSE41258 and GSE68468 dataset. [file 12885_2023_10792_MOESM7_ESM.tif]

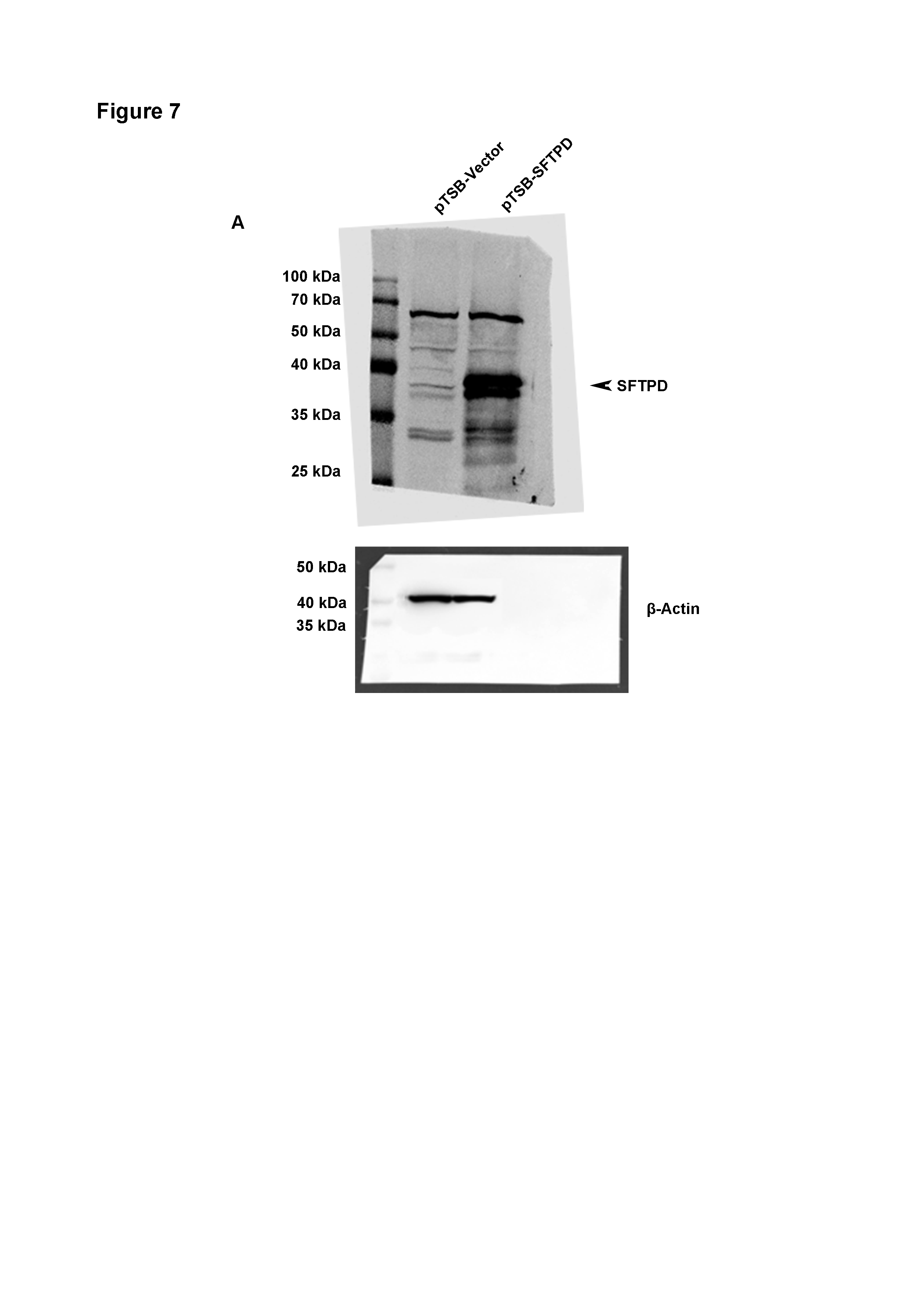

Supplement: Supplementary file 9 — Additional file 9: Origi nal bl ots of Fig. 7. [file 12885_2023_10792_MOESM9_ESM.tif]
